# The Regulation of Insulin-Stimulated Cardiac Glucose Transport via Protein Acetylation

**DOI:** 10.3389/fcvm.2018.00070

**Published:** 2018-06-12

**Authors:** Edith Renguet, Laurent Bultot, Christophe Beauloye, Sandrine Horman, Luc Bertrand

**Affiliations:** ^1^Pole of Cardiovascular Research, Institut de Recherche Expérimentale et Clinique, Université Catholique de Louvain, Brussels, Belgium; ^2^Division of Cardiology, Cliniques Universitaires Saint-Luc, Brussels, Belgium

**Keywords:** glucose uptake, protein acetylation, cardiac metabolism, diabetic cardiomyopathies, insulin resistance

## Abstract

Cellular catabolism is the cell capacity to generate energy from various substrates to sustain its function. To optimize this energy production, cells are able to switch between various metabolic pathways in accordance to substrate availability via a modulation of several regulatory enzymes. This metabolic flexibility is essential for the healthy heart, an organ requiring large quantities of ATP to sustain its contractile function. In type 2 diabetes, excess of non-glucidic nutrients such as fatty acids, branched-chain amino-acids, or ketones bodies, induces cardiac metabolic inflexibility. It is characterized by a preferential use of these alternative substrates to the detriment of glucose, this participating in cardiomyocytes dysfunction and development of diabetic cardiomyopathy. Identification of the molecular mechanisms leading to this metabolic inflexibility have been scrutinized during last decades. In 1963, Randle demonstrated that accumulation of some metabolites from fatty acid metabolism are able to allosterically inhibit regulatory steps of glucose metabolism leading to a preferential use of fatty acids by the heart. Nevertheless, this model does not fully recapitulate observations made in diabetic patients, calling for a more complex model. A new piece of the puzzle emerges from recent evidences gathered from different laboratories showing that metabolism of the non-glucidic substrates induces an increase in acetylation levels of proteins which is concomitant to the perturbation of glucose transport. The purpose of the present review is to gather, in a synthetic model, the different evidences that demonstrate the role of acetylation in the inhibition of the insulin-stimulated glucose uptake in cardiac muscle.

## Introduction

The heart is a muscular pump requiring an enormous quantity of energy to sustain its function. The left ventricle of a human heart ejects 80 ml of blood per heartbeat, the equivalent of a small cup of coffee. However, it beats 75 times per minute. At the end of the day, the human heart will have beaten 100,000 times, ejecting more than 8 tons of blood. This daily work needs 6 kg of ATP, the main cellular energy-providing molecule. This corresponds to 20-times the weight of the heart, which makes it the most energy-consuming organ (per unit weight) of our body (1). The intracellular energy stores (mainly ATP and phosphocreatine) of the heart are negligible. In absence of extracellular substrates, this energy store would be depleted in a few seconds if rescue mechanisms (such as halt of contraction during myocardial ischemia) do not occur. In consequence, the heart has to uninterruptedly produce energy from available nutrients, working on a “just-in-time” basis. This energy production depends on body needs (resting vs. exercise), nutrient availability and hormonal status. In other words, the heart equilibrates energy production to energetic demand and this fine-tuned energetic homeostasis is a prerequisite for optimal cardiac function. Any defect in this equilibrium will have, *de facto*, negative consequences. It is currently recognized that energetic disequilibrium is not just a consequence of various cardiac pathologies but is a trigger in the onset of such pathologies including heart failure (2, 3).

## The metabolic flexibility of the healthy heart

Most of the energy produced by cardiac muscle comes from substrate oxidation in mitochondria. The main substrates used are glucose and fatty acids (1). Fatty acids are preferentially oxidized under fasting and glucose becomes the main ATP-generating metabolite during postprandial state. This metabolic switch has been firstly described by Randle and colleagues in 1963 (4, 5). They have demonstrated that fatty acid oxidation intrinsically inhibits glucose use. The molecular mechanisms involved in this inhibition are partially identified and are a combination of allosteric regulations, phosphorylation events and transcriptional regulations [reviewed in (6, 7)]. First, pyruvate dehydrogenase (PDH), responsible for glucose oxidation, is inhibited by acetyl-coenzyme A (acetyl-CoA) and nicotinamide adenine dinucleotide (NADH), the final products of fatty acid oxidation. They act allosterically on PDH but also activate PDH kinases that phosphorylate and inactivate PDH. Second, acetyl-CoA can be metabolized into citrate. Citrate leaves mitochondria to inhibit 6-phosphosphofructo-1-kinase, the enzyme that controls glycolytic flux. Third, fatty acid oxidation has been shown to directly prevent glucose uptake into myocytes (8, 9). Mechanisms proposed to be involved in glucose uptake inhibition included the impairment of insulin signaling (9) but also acetylation events (summarized in the present review). Next to these short-term mechanisms, fatty acids promote the transcriptional activity of peroxisome-proliferator-activated receptor (PPAR) family. PPARs are transcription factors that increase the expression of genes involved in fatty acid oxidation. On the other hand, the expression of several genes involved in glucose catabolism are decreased by PPARs (2, 10).

Although fatty acids are preferentially used under fasting, glucose becomes the major ATP-providing substrate in postprandial situation, which is characterized by elevated plasma glucose level and insulin secretion. Insulin stimulates glucose uptake, glycolysis, and glucose oxidation whereas it blocks fatty acid oxidation (6). Consequently, the heart constantly shifts from fatty acids to glucose and vice-versa along the day, depending on its nutritional status.

Besides glucose and fatty acids, the heart also uses alternative substrates including ketone bodies, lactate and branched chain amino acids such as leucine (1). Ketone bodies raise in plasma under prolonged fasting. Exercise increases lactate concentration whereas plasma leucine level is elevated under particular diet or during persistent fasting. When elevated, these metabolites counteract the use of other substrates to become the main ATP contributors (11). We can cite the strong and acute inhibition of cardiac glucose uptake by ketone bodies and leucine (12). Similarly, the group of Kieran Clarke has nicely showed that ketone body ester infusion inhibits glycolysis in humans (13).

The multi-faceted metabolic flexibility of the healthy heart allows optimizing energy production, providing the precise amount of ATP required for adequate contractile function. By contrast, cardiac pathologies are generally characterized by metabolic inflexibility, with only one main substrate catabolized to produce energy (2, 3, 14). Hearts of diabetic patients uses quasi exclusively fatty acids, whatever the substrate or hormonal status. In parallel, glucose uptake and catabolism are significantly inhibited. The high level of circulating fatty acids and insulin resistance are two main factors of metabolic inflexibility of the diabetic heart (2).

## Molecular mechanisms regulating glucose uptake

Uptake of glucose, which is the first step of glucose utilization, is inhibited acutely by alternative substrates under physiological conditions but is also blocked under pathological situations such as diabetes. Cardiac glucose transport is principally under the control of glucose transporter 4 (GLUT4) (**Figure 2**). Under basal conditions, GLUT4 is mainly located in intracellular vesicles. Glucose uptake depends on their translocation into sarcolemmal membrane by stimuli such as insulin (6). Insulin promotes the activation of protein kinase B (PKB)/Akt, which can phosphorylate Akt substrate of 160 kDa (AS160). AS160 is the GTPase-activating protein of the G protein Rab that is located on GLUT4 vesicles and responsible for GLUT4 translocation into plasma membrane. The insulin-mediated AS160 phosphorylation inactivates its GAP function, promoting Rab activation. Recent studies strongly suggest that protein acetylation events are, at least partially, involved in the inhibition of glucose transport by ATP-producing substrates such as fatty acids, ketone bodies, and leucine under acute and/or chronic conditions. These studies are summarized in the next chapter.

## Protein acetylation, a post-translational mechanism sensing cellular energetic status

The first observation of an acetylated peptide was made by Narita in 1958 (15, 16). This was followed by others, highlighting amino-terminal acetylation of non-histone proteins. Few years later, the discovery by Phillips (17) of the acetylation of histones on lysine residues by enzymes that were firstly called histone acetyltransferases (HATs) opened a new field of investigation. Several decades later, tubulin was described as the first non-histone protein acetylated on a lysine residue (18).

**Figure 1:**
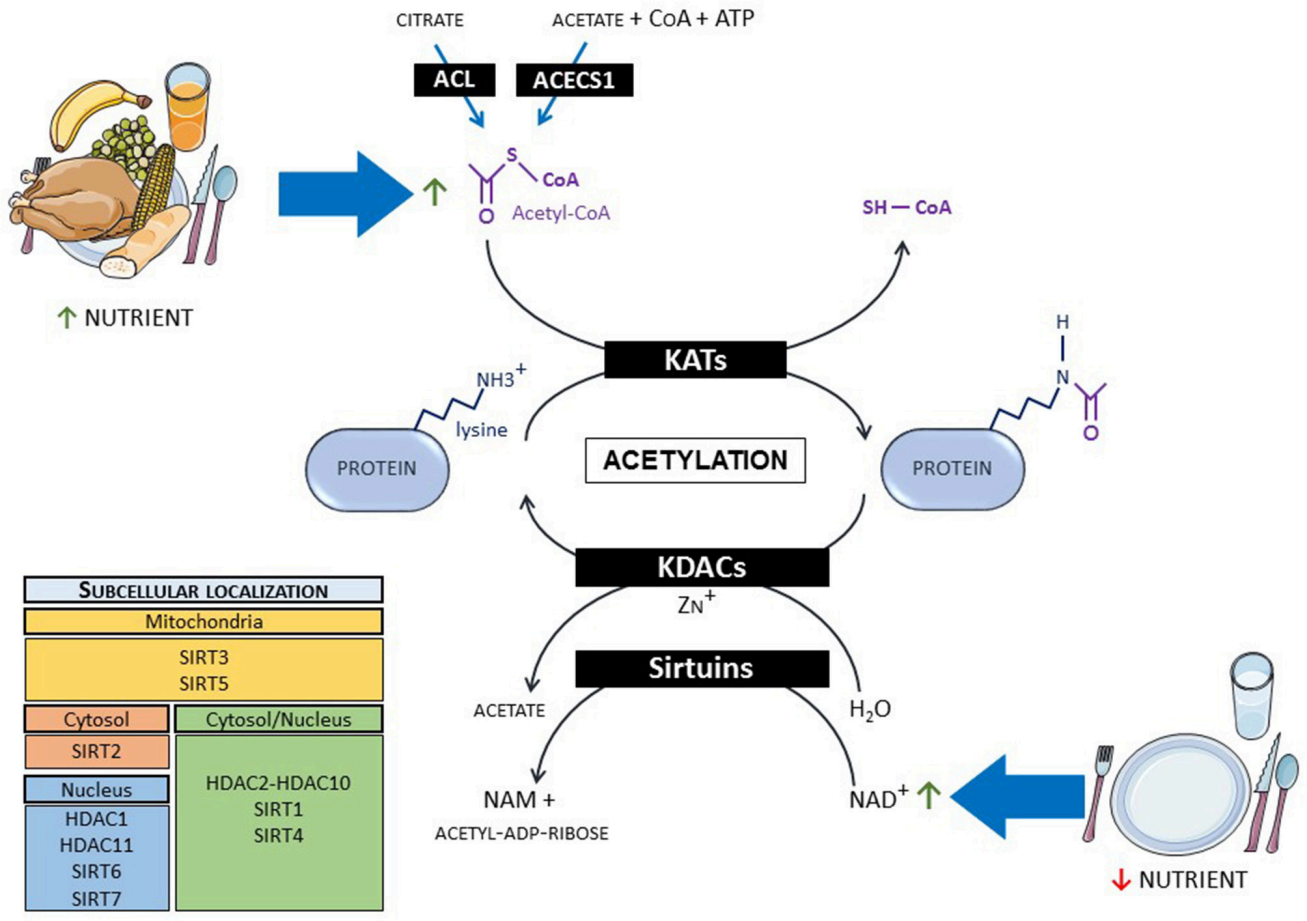
Mechanisms of protein acetylation and link with metabolism. When nutrients are abundant, protein acetylation occurs via the increase in acetyl-CoA level, whereas fasting promotes deacetylation via NAD+ increase. ACL, ATP-citrate lyase; ACECS1, acetyl-CoA synthetase 1; CoA, co-enzyme A; KATs, lysine acetyltransferases; KDACs, lysine deacetylases; NAD, nicotinamide adenine dinucleotide; NAM, nicotinamide.

Despite having a long history, protein acetylation remains a hot topic today with the frequent description of new acetylated proteins. A panel of recent reviews nicely describes the molecular mechanisms involved in this post-translational modification (19–21). To summarize, acetyl group provided by acetyl-CoA is transferred on epsilon-amino group of lysine residues of proteins (histones and non-histones) by spontaneous transfer from acetyl-CoA to proteins (22) or by reactions catalyzed by HAT enzymes that have been renamed lysine acetyltransferases (KATs) (Figure 1). Over 20 different KATs have been identified and can be classified into three major families that share similar structure and function: The general control non-derepressible 5 (GCN5)-related (GNAT) family, the CREB-binding protein (CBP)/p300 family and the MYST family. KATs are mainly localized in the cytoplasm and the nucleus (23). The lack of identified KATs in mitochondria, where more than 60% of the proteins are potentially acetylated, suggests that non-enzymatic acetylation is the main mechanism involved in this organelle (24). Acetylation is a reversible modification with lysine deacetylases (KDACs) catalyzing the deacetylation reaction. Two main families of KDACs were identified and are characterized by different catalytic mechanisms (Figure 1). The first family is composed of Zn^2+^-dependent DACs and are called histone deacetylases (HDAC1 to HDAC11) even if they have multiple substrates including non-histone proteins. The second family is the NAD+-dependent KDACs called sirtuins (SIRT1 to SIRT7). HDAC1, HDAC11, SIRT6, and SIRT7 are localized into the nucleus. SIRT2 is mainly cytoplasmic whereas SIRT3 and SIRT5 are found in the mitochondria. The remaining KDACs shuttle between the nucleus and the cytoplasm (23).

Besides its action in the regulation of gene expression via the regulation of histones, protein acetylation is closely linked to cellular energy status and metabolism via its dependence on acetyl-CoA and NAD+ levels. Indeed, protein acetylation levels are tightly regulated by the availability of acetyl-CoA (20, 25). When available, ATP-generating substrates such as fatty acids, glucose, ketone bodies, and amino acids converge to the mitochondrial production of acetyl-CoA. This acetyl-CoA production enters into the tricarboxylic acid (TCA) cycle but also provokes mitochondrial protein acetylation. The generation of acetyl-CoA in cytoplasm and nucleus mainly depends on the activity of two enzymes, the ATP-citrate lyase (ACL) and the acetyl-CoA synthetase 1 (ACECS1). ACL uses citrate, which increases proportionally to mitochondrial acetyl-CoA, to produce cytosolic and nuclear acetyl-CoA, promoting protein acetylation under fed state. On the other hand, ACECS1 produces acetyl-CoA from acetate, CoA, and ATP, typically in nutrient-restricted conditions. More recently, carnitine acetyltransferase (CrAT) has been proposed to play an important role in cardiac metabolism producing cytoplasmic acetyl-CoA from carnitine and CoA (26). Sirtuins are controlled by cellular NAD+ level. Inasmuch as NADH oxidation gives rise to NAD+, its level fluctuates with respect to the redox state of the cell (21, 27). NAD+ synthesis also depends on its precursor availability, namely nicotinamide, nicotinamide riboside, and nicotinamide mononucleotide.

## Protein acetylation alters glucose uptake

**Figure 2:**
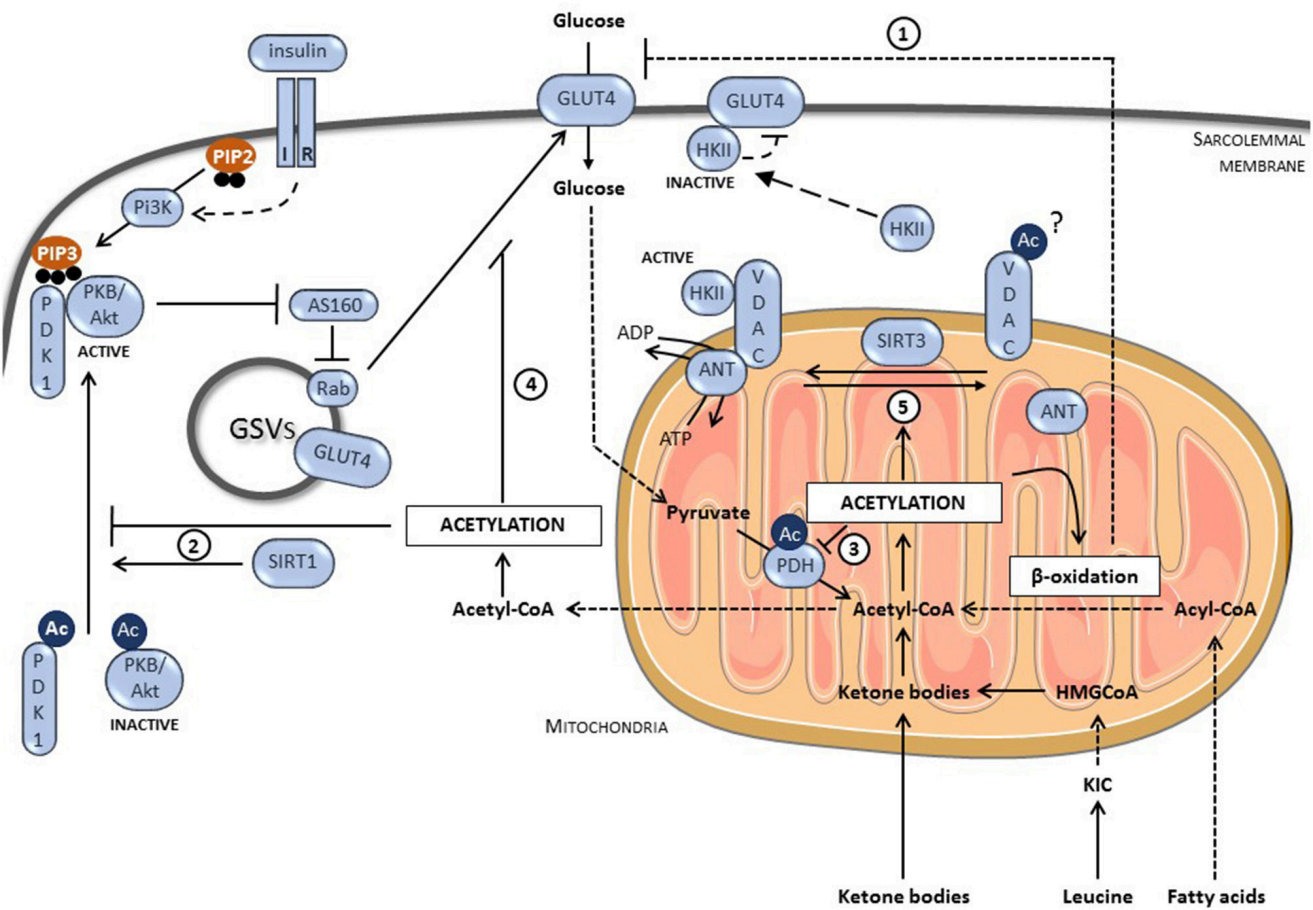
Role of protein acetylation on glucose transport in GLUT4-dependent muscle cells. (1) Protein Acetylation increases fatty acid oxidation, which negatively regulates glucose transport via the mechanisms described by Randle. (2) The deacetylation of PKB/Akt and PDK1 by SIRT1 allows their interaction with PIP3 and subsequent activation. In consequence, protein acetylation limits insulin signaling and reduces glucose uptake. (3) Protein acetylation inhibits glucose oxidation, inducing accumulation of glycolytic intermediates that could block glucose uptake. (4) Protein acetylation inhibits GLUT4 translocation by a still unknown mechanism. (5) Protein acetylation prevents the formation of the VDAC/ANT complex required for HKII binding and activation. Inactive HKII promotes the accumulation of intracellular glucose that inhibits GLUT4. IR, insulin receptor; PI3K, phosphatidyl-inositol-3 kinase; PIP2, phosphatidylinositol (4,5)-trisphosphate; PIP3, phosphatidylinositol (3,4,5)-trisphosphate; GLUT4, glucose transporter 4; PKB/Akt, protein kinase B; PDK1, phosphoinositide-dependent kinase 1; AS160, Akt substrate of 160 kDa; GSVs, GLUT4 storage vesicles; HKII, hexokinase II; VDAC, voltage-dependent anion channel; ANT, anion nucleotide transporter; SIRT3, sirtuin 3; SIRT1, sirtuin 1; KIC, α-ketoisocaproate; HMGCoA, β-Hydroxy β-methylglutaryl-CoA; Ac, acetylated-lysine.

Keeping in mind the tight interconnection between acetylation and energy status, numerous studies evaluated the control of metabolism by acetylation processes, including in the heart. Fukushima and Lopaschuk recently summarized the impact of protein acetylation on cardiac fatty acid metabolism, particularly in diabetes where mitochondrial acetylation of numerous metabolic enzymes (including long chain acyl-CoA dehydrogenase, β-hydroxyacyl CoA dehydrogenase, carnitine palmitoyl transferase-2, and CrAT) participates in the chronic increase in fatty acid oxidation (21, 27). This increase in fatty acid oxidation will decrease glucose uptake and metabolism via Randle mechanisms explained above (Figure 2). Besides fatty acid metabolism, protein acetylation takes a growing importance in the direct regulation of glucose metabolism. In 2005, Rogers and colleagues demonstrated that fasting enhanced SIRT1 expression and activity, inducing deacetylation of the transcriptional factor peroxisome proliferator-activated receptor-γ coactivator 1α (PGC1-α), responsible for the induction of gluconeogenic genes and the repression of glycolytic genes in liver (28). Shortly afterwards, several studies have shown that SIRT1 activation with small molecules such as SRT1720 and resveratrol improved glycaemia of diabetic rodent models (29, 30). This was associated with a better glucose disposable rate, reflecting an improvement of glucose uptake in muscle (29, 30). The beneficial effects observed in diabetic models by activation of KDACs were largely described to be mediated via a regulation of key transcriptional factors (including PGC1-α and forkhead box O) involved in regulation of mitochondrial biogenesis and expression of key metabolic enzymes (31). Interestingly, several high-throughput mass-spectrometry studies revealed that, besides histones and transcriptional regulators, numerous enzymes involved in metabolism were acetylated (32–35). Impact of the majority of these acetylation events on metabolism remains currently unknown. Current research starts to identify which ones are meaningful and how they will influence cellular metabolism.

In this mini-review, we particularly focus our interest on the impact of acetylation on insulin-stimulated glucose uptake in the heart, but also depict related studies performed in muscle and other tissues. To summarize, protein acetylation can potentially affect glucose uptake by directly targeting glucose transport but also by regulating the insulin signaling pathway or by acting on glucose oxidation (Figure 2). Concerning the insulin pathway, both PKB/Akt and its upstream regulator phosphoinositide-dependent kinase 1 (PDK1) were shown to be acetylated on lysine residues located in their pleckstrin homology (PH) domain. Acetylation of PH domains interfered with their binding to phosphatidylinositol (3,4,5)-trisphosphate (PIP3), a crucial step for PKB/Akt activation (36) (Figure 2). SIRT1 can deacetylate PKB/Akt and PDK1, enhancing their binding to PIP3 and therefore, increasing insulin signaling (36). Accordingly, SIRT1 deficient mice present a higher PKB/Akt acetylation level in the heart, affecting its activation in response to hypertrophic stimulus (36). PKB/Akt acetylation is also increased in heart of mice undergoing a high fat diet (HFD) (37). This increase correlates with a lower expression of SIRT3 and a lower activation of PKB/Akt. In adipose tissues, SIRT2 forms a complex with PKB/Akt under nutrient deprivation. Once complexed, SIRT2 can reduce PKB/Akt acetylation promoting its phosphorylation/activation after insulin stimulation (38). Upstream of PKB/Akt, SIRT1 can deacetylate the insulin receptor substrate-2 (IRS-2), which links the activated insulin receptor to its downstream effectors (39). Deacetylation of IRS2 promotes its phosphorylation on tyrosine residues and its activation.

Several studies showed that increase in acetylation correlates with a reduction in glucose oxidation and/or glycolysis, leading to an accumulation of metabolic intermediates, resulting *in fine* in glucose uptake inhibition (Figure 2). Strong evidences are described for a control of PDH by acetylation. Mori et al. have revealed the presence of an increased acetylation level of PDH in hypertrophic hearts (40). This PDH acetylation, which is due to a reduction in SIRT3 expression, correlates with a decrease in its activity and in glucose oxidation. Similar observations were made in muscle cells (41). Horton and collaborators have shown that succinate dehydrogenase (SDHA), a protein involved in TCA cycle and member of the respiratory complex II, was acetylated on Lys-179 in the failing heart (42). This acetylation in the FAD+ binding-region decreased SDHA activity, resulting in succinate accumulation and a reduced complex II-driven respiratory rate. SIRT3 also deacetylates various members of the mitochondrial respiratory complex I and SIRT3-deficient cells presented a lower complex 1-driven respiratory rate (43). Taken together, these results show that a global increase in mitochondrial protein acetylation will decrease TCA cycle and electron transport chain activities in heart resulting in a lower capacity to oxidize glucose, accumulating intermediates of glucose catabolism that could finally decrease glucose uptake. However, the relative implication of all these acetylation processes in the acute regulation of cardiac glucose metabolism under physiological state (Randle effect) and/or in its chronic inhibition in diabetes (metabolic inflexibility) remain largely unknown.

Finally, protein acetylation directly affects glucose uptake via its action on GLUT4 (Figure 2). Our laboratory has recently proposed that a global increase in protein acetylation by leucine or ketone bodies (two acetyl-CoA-providing metabolites), in primary cultured cardiomyocytes and *ex vivo* perfused hearts, decreases the insulin-stimulated glucose transport via a blockage of GLUT4 translocation to the cell membrane (12). The exact mechanisms responsible for the defective GLUT4 translocation remain unknown but give another clue in the loss of glucose utilization by the diabetic heart, inasmuch as the plasmatic level of both leucine and ketone bodies is increased in diabetic patients. Lantier and colleagues have also linked the acetylation-dependent decrease of muscle glucose transport to an inhibition of hexokinase II (HKII), the enzyme responsible for glucose phosphorylation after its uptake (44). Indeed, in order to be fully activated, HKII binds the mitochondrial permeability transition pore (mPTP) composed of the voltage-dependent anion channel (VDAC) located in the outer membrane and the anion nucleotide transporter (ANT) (45). In muscle from SIRT3-deficient mice, especially under HFD, the interaction between mPTP and HKII as well as between VDAC and ANT were weaker, resulting in lower HK activity, in intracellular glucose accumulation and, subsequently, to a reduction in glucose uptake. This supports the model showing that HKII interaction with its partners is important for its activity (46).

In contrast to all these reports, it must be mentioned that another study proposed a model where the acetylation of a protein called TUG releases GLUT4 storage vesicles (GSVs) from the Golgi apparatus. This promotes GSV translocation to the plasma membrane and glucose uptake (47). An explanation of such discrepancy could be linked to the fact that this study has been performed in adipocytes whereas most of the studies presented in this review have been performed in muscle. It will be highly interesting to study the role of TUG acetylation in GLUT4 translocation in muscle.

## Conclusions

Except the last finding, there is a large consensus indicating that an increase in protein acetylation diminishes cardiac glucose uptake after insulin stimulation. Such event could occur under physiological condition when non-glucidic substrate levels increase in plasma, but could also participate in the establishment of cardiac metabolic inflexibility in pathologies. Fatty acids and leucine, which are both early markers of diabetes (48), induce acetyl-CoA accumulation and protein acetylation (12, 37), suggesting that acetylation could be an early event in insulin resistance development. This makes protein acetylation a valuable target for new treatment of diabetes. Acetylation level could be controlled by affecting the activity or the expression of KDACs and KATs. Therefore, a tight control of the activity of these enzymes could prevent, reverse or, at least, delay the establishment of the metabolic inflexibility in the diabetic heart. This could be achieved by a modulation, via dietary or pharmaceutical intervention, of NAD+ and/or acetyl-CoA levels (20, 27, 49). Even if we can conclude that protein acetylation is definitely a crucial actor in the apparition of metabolic inflexibility in the heart during diabetes, numerous questions remain unanswered. Future research will allow us to fully understand the mechanisms involved in the deregulation of acetylation events and to assess its validity as a target for diabetic treatment.

## Author contributions

ER, LaB, CB, SH, and LuB wrote and edited the present review.

## Conflict of interest statement

The funders had no role in study design, data collection and analysis, decision to publish, or preparation of the manuscript. All authors declare no conflict of interest. The reviewer SK declared a past co-authorship with one of the authors to the handling Editor.
