# Erratum: The Regulation of Insulin-Stimulated Cardiac Glucose Transport via Protein Acetylation

**DOI:** 10.3389/fcvm.2018.00103

**Published:** 2018-07-12

**Authors:** Frontiers Production Office

**Affiliations:** Frontiers Production Office, Frontiers Lausanne, Switzerland

**Keywords:** glucose uptake, protein acetylation, cardiac metabolism, diabetic cardiomyopathies, insulin resistance

In the original article, the reference list was incorrectly numbered and this resulted in mismatched citations. This was fixed and the original article was updated accordingly.

The publisher apologizes for this mistake.

The original article has been updated.

